# Development of Maxillary Sinuses in Girls and Boys from Birth to Age 18 Based on Computed Tomography Investigation

**DOI:** 10.3390/diagnostics16132051

**Published:** 2026-06-30

**Authors:** Przemysław Kiciński, Piotr Grzelak, Beata Małachowska, Michał Podgórski, Michał Polguj

**Affiliations:** 1 Department of Angiology, Chair of Anatomy and Histology, Medical University of Lodz, 90-419 Lodz, Poland; 2 Department of Diagnostic Imagining, Polish Mother’s Memorial Hospital Research Institute, 93-338 Lodz, Poland; piotr.grzelak@iczmp.edu.pl; 3 Department of Radiation Oncology, Albert Einstein College of Medicine, Bronx, NY 10461, USA; beata.malachowska@einsteinmed.org; 4 III Department of Radiology and Diagnostic Imaging, Medical University of Lodz, 90-419 Lodz, Poland; michal.podgorski@umed.lodz.pl; 5 Department of Normal and Clinical Anatomy, Chair of Anatomy and Histology, Medical University of Lodz, 90-419 Lodz, Poland; michal.polguj@umed.lodz.pl

**Keywords:** maxillary sinus, development, children, correlation, computed tomography

## Abstract

**Background/Objectives:** Maxillary sinuses play an important clinical role due to their location within the visceral cranium and proximity to numerous anatomical structures. In the case of children, considering the ongoing development process, it should be remembered that they are characterized by great variability in both size and shape. The aim of this study was to analyze the development of the right and left maxillary sinuses in relation to the age of children both in the study group and independently in girls and boys. **Methods:** This study was retrospective in nature. It was based on the results of head computed tomography examinations. The study group included 468 children from birth to the age of 18. To assess the development of the maxillary sinuses, the correlation between precisely defined measurements of the right and left maxillary sinuses and age was analyzed. **Results**: An analysis of the relationship between specific dimensions of the maxillary sinuses and the age of children revealed a very strong positive correlation for volume and height (r > 0.9, *p* < 0.0001) and a strong correlation for length and width (r > 0.8, *p* < 0.0001). The correlation for individual dimensions was similar in girls and boys. Both in the study group and for girls and boys, the correlation was symmetrical—similar for the right and left maxillary sinuses. The values of the correlation coefficients for the volume that assesses the growth of the maxillary sinuses in all directions were r = 0.92 and r = 0.91 in girls, and r = 0.91 and r = 0.92 in boys for the right and left maxillary sinus, respectively. **Conclusions:** A statistically significant positive correlation was found between the development of the maxillary sinuses and the age of children. The increase in the volume of the right and left maxillary sinuses was close to linear from birth to 15–16 years of age in girls and 17 years of age in boys.

## 1. Introduction

The maxillary sinus is the largest paranasal sinus, which is a substantial space located inside the maxilla. Development of the maxillary sinuses begins in utero and continues after birth [1,2,3,4]. As a result of pneumatization of the maxilla, maxillary sinuses become increasingly larger in the subsequent years of life. This results in a reduction in the amount of bone in the maxilla, and the walls of the maxillary sinuses become thinner and more delicate. The location of the maxillary sinuses within the viscerocranium and their proximity to numerous anatomical structures is of clinical importance. Maxillary sinuses are adjacent to, among other things, the nasal cavity, the orbit, the pterygopalatine fossa, the infratemporal fossa, the oral cavity and the anatomical structures located within them. Moreover, depending on the degree of maxillary pneumatization, some anatomical structures may be located in the lumen or remain in the immediate vicinity of the maxillary sinuses [1,3,5]. Knowledge of the development and structure of the maxillary sinuses is important in many fields of medicine. In the case of children, it is important to bear in mind that they show great variability in both size and shape in subsequent years of life. This knowledge is important, for example, when planning endoscopic or surgical procedures in children. It may allow for better understanding of the effects of diseases related to maxillary sinuses or anatomical structures located in their immediate or indirect vicinity. It also plays an important role in cases of viscerocranium injuries. The degree of maxillary pneumatization also plays a role in the spread of pathological processes from maxillary sinuses to adjacent areas and anatomical structures and vice versa [1,2,3,6].

Therefore, we decided to conduct a comprehensive analysis of the development of the maxillary sinuses, analyzing the right and left maxillary sinuses in both girls and boys, considering the following measurements: volume, height, width, and length.

The aim of this study was to analyze the relationship between the defined measurements of the right and left maxillary sinuses and the age of children from birth to 18 years of age, both in the study group and independently in girls and boys.

## 2. Materials and Methods

The study comprised a group of 468 children, aged from birth to 18 years, who underwent computed tomography (CT) of the head and met the inclusion criteria. Due to the different rates of development of paranasal sinuses in subsequent periods of life, 234 girls and 234 boys were qualified for the study. The number of children in each year of life was the same (26, 13 girls and 13 boys). The inclusion criterion was a properly performed head CT scan. The exclusion criteria were as follows: artifacts that make assessment of the skull and paranasal sinuses impossible, disease of the paranasal sinuses or pathology within them, history of growth disorders, traumatic cranial bone and head changes, history of head surgery, cranial deformation, genetic or metabolic disease, congenital or acquired developmental defect, and active neoplastic process [7,8].

This study, conducted at the Department of Diagnostic Imaging, Polish Mother’s Memorial Hospital—Research Institute, was retrospective in nature. All examinations were performed using a 256-row Philips Brilliance computed tomography scanner (Philips Healthcare, Best, The Netherlands). The acquisition protocol included a tube voltage of 120 kV and a tube current of 50 mA, with a scan range of 240 mm. Additional acquisition parameters included a 270° scan angle and an outward scan direction (Direction OUT). Informed consent was obtained from parents/legal guardians for the children’s head computed tomography scans to be used in this study.

**Figure 1:**
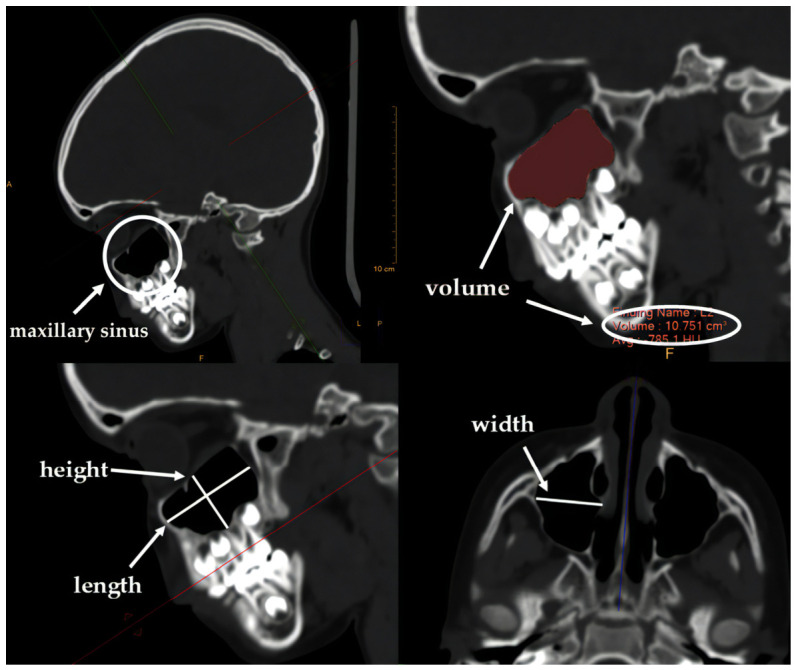
Head computed tomography in children showing the maxillary sinus measurements used in the study: volume, height, length, and width.

For the children included in this study, bilateral assessment of the maxillary sinuses was performed in three planes, namely, the sagittal, coronal (frontal), and transverse planes. All measurements were the maximum internal dimensions of a given maxillary sinus and were taken in the bone window. The maximum vertical diameter of the sinus perpendicular to the transverse plane was defined as the maxillary sinus height. The maximum anteroposterior diameter of the sinus perpendicular to the frontal plane was defined as the maxillary sinus length. The maximum transverse diameter of the sinus perpendicular to the sagittal plane was defined as the width of the maxillary sinus. The volume of the right and left maxillary sinuses was also measured in all children. Maxillary sinus volume measurements were performed using IntelliSpace Portal 7.0 with the “Tumor Tracking” option (Figure 1).

Statistical analysis: The mean (M) and standard deviation (SD), along with the 95% confidence interval (CI), were used to present descriptive statistical values for continuous variables. Nominal variables were described as counts. Student’s *t*-test was used to compare continuous variables between two groups. Paired student’s test was used to compare measurements between left and right side. The correlations between variables were measured by Pearson correlation. The Pearson correlation coefficient was described according to the following division: 0.0–0.3—no/weak correlation; 0.31–0.5—moderate; 0.51–0.7—high; 0.71–0.9—strong; 0.91–1.0—very strong. Correlations between selected variables demonstrate scatter plots. Statistical analysis was performed using STATISTICA 13.3 (TIBCO Software, Palo Alto, CA, USA). A *p* value < 0.05 was considered statistically significant.

## 3. Results

In the study group of 468 children, the mean age was 9.01 ± 5.20 years (95% Cl, 8.54–9.49). The mean age of girls was 9.03 ± 5.22 years (95% Cl, 8.36–9.70), whereas that of boys was 9.00 ± 5.20 years (95% Cl, 8.33–9.67) (*p* = 0.9506). The analysis included 468 right and 468 left maxillary sinuses, respectively. No statistically significant difference was observed between the analyzed measurements of the right and left maxillary sinuses, both in the study group and in girls and boys (Table 1 and Table 2).

Table 2 shows the mean volume, height, length, and width of the right and left maxillary sinuses in girls and boys. When analyzing maxillary sinuses in girls and boys, no statistically significant differences were found for both the right (volume, *p* = 0.3321; height, *p* = 0.9161; length, *p* = 0.1901; width, *p* = 0.2100) and left (volume, *p* = 0.2146; height, *p* = 0.7797; length, *p* = 0.1342; width, *p* = 0.3470) maxillary sinuses.

**Figure 2:**
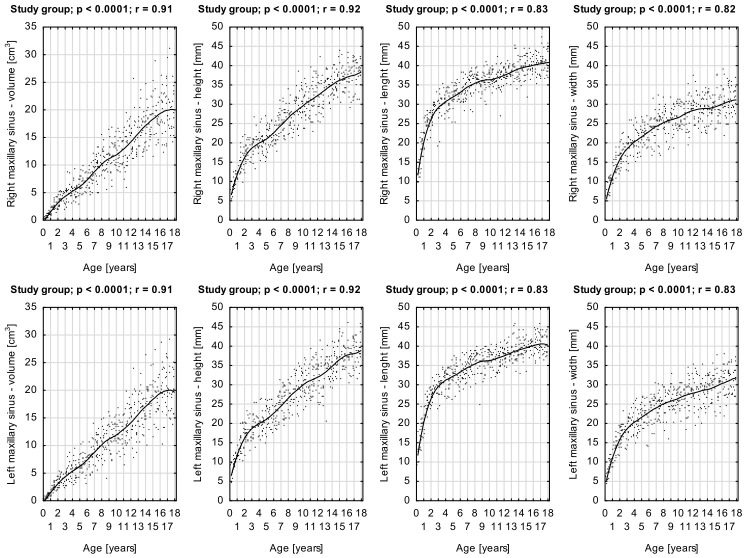
Graphs of the correlations between the measurements of the right and left maxillary sinuses and age in the study group. The Pearson correlation coefficient (r) is provided; *p* < 0.0001 for all analyzed correlations.

**Figure 3:**
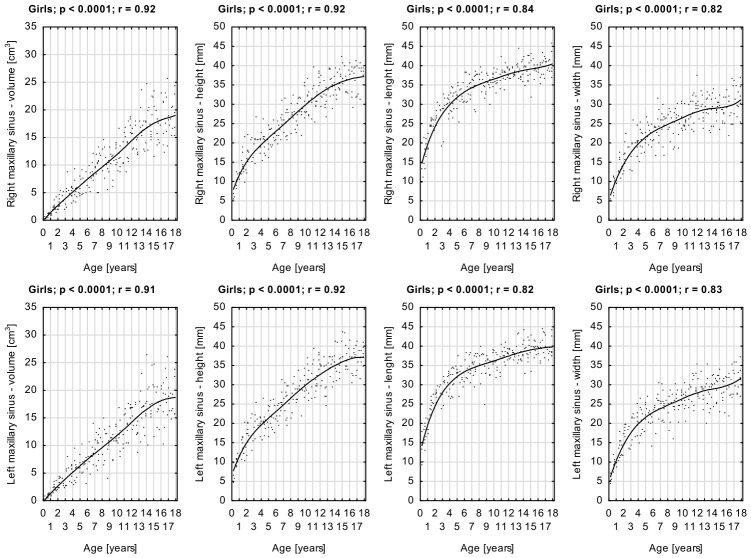
Graphs of the correlations between the measurements of the right and left maxillary sinuses and age in girls. The Pearson correlation coefficient (r) is provided; *p* < 0.0001 for all analyzed correlations.

**Figure 4:**
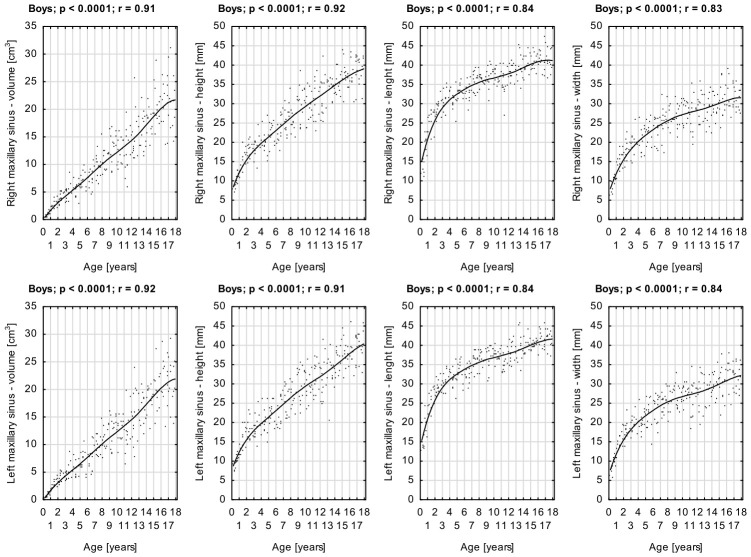
Graphs of the correlations between the measurements of the right and left maxillary sinuses and age in boys. The Pearson correlation coefficient (r) is provided; *p* < 0.0001 for all analyzed correlations.

Both in the study group and in girls and boys, a statistically significant positive correlation was detected at a significance level of *p* < 0.0001 between the age of the children and all analyzed measurements of the right and left maxillary sinuses. There was a very strong correlation between age and the volume and height of right and left maxillary sinuses, both in the study group and separately in girls and boys (r > 0.9, *p* < 0.0001). However, the correlation between age and the length and width of right and left maxillary sinuses was strong in the study group and separately in girls and boys (r > 0.8, *p* < 0.0001). The values of the correlation coefficients for the volume that assesses the growth of the maxillary sinuses in all directions were r = 0.92 and r = 0.91 in girls, and r = 0.91 and r = 0.92 in boys, for the right and left maxillary sinus, respectively. The correlation for each dimension was similar in girls and boys. For both girls and boys, the correlation was symmetrical—similar for the right and left maxillary sinuses. Detailed values of the Pearson correlation coefficient for all analyzed relationships are given in Figure 2, Figure 3 and Figure 4.

## 4. Discussion

In this study, we demonstrated a strong and very strong positive correlation between the analyzed measurements of the right and left maxillary sinuses and the age of girls and boys from birth to age 18. There are studies available in the literature that confirm the correlation between the development of maxillary sinuses and age [2,6,9,10,11,12,13]. According to some authors, the postnatal development of maxillary sinuses in children occurs in two phases. However, some authors disagree with this model, arguing that after an initial period of rapid growth, there is further slow growth of maxillary sinuses [2,6]. Bhushan et al. analyzed maxillary sinus measurements in three age groups: up to 6 years, 6 to 12 years, and over 12 years. They showed that the increase in the length and width of the maxillary sinuses in the first two age groups was statistically significant, whereas in the last age group, it was statistically insignificant. For height and volume measurements, the authors observed that the increase was statistically significant in all age groups [11]. A similar pattern of maxillary sinus growth in magnetic resonance imaging studies was described by Barghouth et al. [10]. Among height, length, and width, Lee et al. observed that the height of the maxillary sinuses increased for the longest time [14]. Lorkiewicz-Muszyńska et al. reported faster growth in the first few years of life for width, height, and length, with a slower growth in the following years. However, regarding the volume, both for girls and boys, they found a constant, almost linear increase until the age of 14 [9]. Sarilita et al. analyzed the volume of the maxillary sinuses in a group of patients up to 25 years of age, divided into five age groups. They also showed a practically linear increase in volume up to the age group of 16–20 years [15]. The data presented in our study show that the correlation coefficient for the length and width was lower than for the height and volume of the maxillary sinuses. Based on the scatterplots we presented, it can be observed that the growth of the maxillary sinuses in length and width was fastest in the first few years of life. Then, in the following years of life, a gradual slowdown in growth is seen in length and width. For the height measurement, the slowdown in growth in the subsequent years was smaller than in the case of length and width. However, in the case of volume, an almost linear increase in the volume of the right and left maxillary sinuses can be observed until approximately 15–16 years of age in girls and 17 years of age in boys. It should be noted that the volume assesses the growth of the maxillary sinus in all directions and is not just the greatest diameter in a specific axis. The growth of the right and left maxillary sinuses after birth for all analyzed measurements was similar and symmetrical in both girls and boys.

Regardless of the growth model, authors typically agree that the development of the maxillary sinuses is observed in the prenatal period and during the first dozen or so years of postnatal life. However, there is no such agreement when determining the age until which maxillary sinuses continue to grow. According to various publications, this is most often the period from about 15 to 20 years of age [9,11,13,15,16]. However, Jun et al. indicate that the volume of the maxillary sinuses increases until the fourth decade of life in men and until the third decade of life in women [17]. Lee et al., who analyzed a group of children up to 15 years of age, reported that, depending on the size of the maxillary sinus, growth continued until the age of 11–13 years [14]. Bhushan et al. and Vaid et al., in their studies, draw attention to the occurrence of slow pneumatization of the maxillary sinus, which can last up to 20 years of age [11,16]. The reports of most authors regarding the duration of the maxillary sinus growth process are also confirmed by our study. It is documented in the literature that changes in the shape of the maxillary sinuses and the skull continue throughout life [4,15,17]. Presenting a comprehensive analysis of the growth of the right and left maxillary sinuses in girls and boys is important as the growth pattern may depend on many factors, such as genotypic or phenotypic factors.

There is consensus among researchers that the average size of the maxillary sinuses is smaller in girls than in boys. In the studies by Barghouth et al., Bhushan et al., Lorkiewicz-Muszyńska et al., and Park et al., maxillary sinuses in girls are smaller than in boys, and no statistically significant difference was found [9,10,11,13]. In the study by Adibelli et al., who analyzed 1383 children, maxillary sinuses were also smaller in girls than in boys, and this difference was statistically significant [12]. In our study, the mean values of the individual dimensions of the maxillary sinuses were smaller in girls than in boys. They were not statistically significant for either the right or the left maxillary sinuses. We compared the mean values of the individual maxillary sinus dimensions of girls and boys. The number of girls and boys in each age group was the same. The development of the maxillary sinuses correlated with the development of the craniofacial skeleton [5,7]. Kiciński et al. also showed a statistically significant positive correlation with the development of the cranium [7]. However, the average cranial size was smaller in girls than in boys [7,18,19].

In studies conducted on children, in which the development of the maxillary sinuses was assessed, and measurements such as height, length, width, and volume were analyzed, no statistically significant difference was found between the right and left maxillary sinuses [9,10,11,12]. Park et al. and Değermenci et al., analyzing the development of paranasal sinuses based on volume measurements, also found no statistically significant difference between the volume of the right and left maxillary sinuses [13,20]. Therefore, in many publications, the authors perform a collective analysis of the maxillary sinuses without dividing them into right and left. It should be remembered that the presented data concerns the entire group of patients. However, maxillary sinuses are not identical in individual patients. Hypoplasia is an extreme example of a situation where a large difference in the size of the maxillary sinuses is observed. Maxillary sinus hypoplasia may be unilateral or bilateral. It should also be remembered that in the case of unilateral maxillary sinus hypoplasia, the size of one of the sinuses is significantly smaller, whereas the maxillary sinus on the other side is of normal size [6,21,22,23,24,25]. In our study, no statistically significant difference was found between the analyzed dimensions of the right and left maxillary sinuses. Since humans have two maxillary sinuses, for a comprehensive assessment, an independent analysis is performed of the right and left maxillary sinuses in girls and boys.

A certain limitation of this study is its analysis of participants up to the age of 18. There is a lack of analysis of young adults. Furthermore, we did not establish centile charts because, to create them, a sufficiently large number of head computed tomography scans in girls and boys must be analyzed to investigate maxillary sinuses. The greater the number of studies in each age range, the more reliable the percentile charts. It is believed that in each age group, there should be at least several dozen—and preferably several hundred—results for girls and boys, respectively.

## 5. Conclusions

This study provides a comprehensive description of the development of maxillary sinuses in children. We demonstrated a positive correlation between maxillary sinus growth and age in girls and boys. The increase in the volume of the right and left maxillary sinuses is close to linear from birth to 15–16 years of age in girls and 17 years of age in boys. However, the maxillary sinuses grew most rapidly in height, length, and width in the first years of life. At the same time, we observed a smaller slowdown in the growth of height than in that of length and width in the subsequent years of life.

## Figures and Tables

**Table 1:** Measurements of the right and left maxillary sinus in the study group.

Maxillary Sinus Measurement	Group (*n*-468) Mean ± SD (95% Cl)		
	Right Maxillary Sinus	Left Maxillary Sinus	*p* *
Volume [cm^3^]	10.97 ± 6.58 (10.37–11.56)	10.98 ± 6.58 (10.38–11.58)	0.8118
Height [mm]	26.95 ± 8.85 (26.15–27.76)	27.02 ± 8.96 (26.20–27.83)	0.4767
Length [mm]	34.17 ± 6.70 (33.57–34.78)	34.15 ± 6.58 (33.55–34.75)	0.7574
Width [mm]	24.20 ± 6.80 (23.58–24.82)	24.12 ± 6.79 (23.50–24.74)	0.4211

*p* *—right maxillary sinus vs. left maxillary sinus.

**Table 2:** Measurements of the right and left maxillary sinus in girls and in boys.

Maxillary Sinus Measurement	Girls (*n*-234) Mean ± SD (95% Cl)			Boys (*n*-234) Mean ± SD (95% Cl)		
	Right Maxillary Sinus	Left Maxillary Sinus	*p* *	Right Maxillary Sinus	Left Maxillary Sinus	*p* *
Volume [cm^3^]	10.67 ± 6.29 (9.86–11.48)	10.60 ± 6.30 (9.79–11.41)	0.3642	11.26 ± 6.85 (10.38–12.14)	11.36 ± 6.83 (10.48–12.24)	0.2709
Height [mm]	26.91 ± 8.84 (25.77–28.05)	26.90 ± 8.91 (25.75–28.05)	0.9266	27.00 ± 8.88 (25.85–28.14)	27.13 ± 9.03 (25.97–28.30)	0.3210
Length [mm]	33.77 ± 6.78 (32.89–34.64)	33.70 ± 6.68 (32.83–34.56)	0.4826	34.58 ± 6.60 (33.73–35.43)	34.61 ± 6.47 (33.77–35.44)	0.7859
Width [mm]	23.80 ± 6.86 (22.92–24.69)	23.83 ± 6.91 (22.94–24.72)	0.8618	24.59 ± 6.73 (23.73–25.46)	24.42 ± 6.68 (23.56–25.28)	0.2024

*p* *—right maxillary sinus vs. left maxillary sinus.

## Data Availability

The data that support the findings of this study are available from the corresponding author: Przemysław Kiciński, e-mail: przemyslaw.kicinski@umed.lodz.pl or kicinskiprzemko@gmail.com.
